# Interpersonal Relationship Stress Brings on Social Networking Sites Addiction Among Chinese Undergraduate Students

**DOI:** 10.3389/fpsyg.2022.905971

**Published:** 2022-06-22

**Authors:** Bi Li, Kaihui Zhang, Yan Wu, Zhifeng Hao

**Affiliations:** ^1^Laboratory for Language Engineering and Computing, Guangdong University of Foreign Studies, Guangzhou, China; ^2^School of Business, Guangdong University of Foreign Studies, Guangzhou, China; ^3^School of Information Science and Technology, Guangdong University of Foreign Studies, Guangzhou, China; ^4^Department of Applied Psychology, Guangdong University of Foreign Studies, Guangzhou, China; ^5^College of Science, Shantou University, Shantou, China

**Keywords:** sequential mediation, interpersonal relationship stress, WeChat addiction, WeChat use intensity, WeChat social interaction

## Abstract

The adverse effects of life stress on social networking sites addiction are increasingly recognized, but so far there is little evidence on how and which specific types of life stress are conducive to the addictive behavior. Interpersonal relationship stress being the main source of stress for undergraduates, the purpose of the current paper is thus to delve into whether perceived stress in interpersonal relationships significantly leads to WeChat addiction and, if so, how this type of stress drives the excessive use of WeChat. The data was collected from self-report questionnaires completed by 463 Chinese undergraduate students and then analyzed with structural equation modeling. The results revealed that the positive association between WeChat users’ interpersonal relationship stress and addictive behavior is fully and sequentially mediated by WeChat use intensity and social interaction. More specifically, accumulation of stress in interpersonal relationships gives rise to the intensity of WeChat use, which in turn fuels rising addiction to WeChat both directly and indirectly via social interaction on WeChat. These findings contribute to a more refined understanding of the pathological use of WeChat.

## Introduction

Social networking sites (SNS) serve as popular tools for increasing users’ social capital and facilitating communication (Carbonell and Panova, 2017). In addition, the spread of smartphones promotes the growth in SNS use and addiction, whose detrimental outcomes attract growing scholarly attention (Yang et al., 2016). For instance, Facebook is one of the most popular SNS and has thus been the subject of much research; some researchers have empirically identified the adverse consequences of Facebook addiction, such as decreasing life satisfaction (Błachnio et al., 2016) and increasing anxiety (Fox and Moreland, 2015). WeChat is the most popular SNS in China, whose global monthly active users has been increasing steadily to over 1.26 billion in the third quarter of 2021 (Statista, 2021). Empirical evidences have also shown the significant negative effects of addictive behavior on WeChat users’ physical, mental, and social health (Xue et al., 2018). Moreover, magnetic resonance imaging data demonstrate that WeChat addiction reduces gray matter volumes within the subgenual anterior cingulate cortex of addicts’ brains (Montag et al., 2018).

Addiction is one category of neuropsychiatric disorder, characterized by pathological behavior for which no treatment exists (Creed, 2017). Similarly, there is no well-documented cure for SNS addiction specifically (Andreassen and Pallesen, 2014). Determining antecedents and dissecting mechanisms of SNS addiction are crucially important for advancing our understanding of this condition and for the development of effective interventions and therapies. It is not surprising, therefore, researchers have been eager to investigate the underlying causes of undergraduate users’ SNS addiction, such as personality traits (Tang et al., 2016), and interpersonal issues (Andreassen and Pallesen, 2014). Young adult’s stress is a significant psychology and health issue associated with microblog use (Feng et al., 2019), and can induce Internet addiction (Yan et al., 2014). The adverse influences of SNS on this demographic group deserve close attention in any nation (Tandon et al., 2021). Researchers have demonstrated a link between undergraduate users’ life stress and WeChat addiction (Li et al., 2018), however the underlying mechanisms have yet to be unpicked. Moreover, how and which specific types of stressful life events significantly contribute to the addictive behavior, remain poorly understood. Interpersonal relationship problems constitute one of the most significant sources of stress for undergraduates (Chiu, 2014). In this study, therefore, we aim to delve into whether interpersonal relationship stress is significantly associated with WeChat addiction among undergraduates and, if so, how this type of stress drives WeChat addiction.

Compensatory Internet use theory suggests that people go online to escape real life issues or alleviate dysphoric moods (Kardefelt-Winther, 2014). Thus, internet addiction has been considered as a behavioral response to life stress (Li et al., 2016). Facebook is particularly attractive for users experiencing this kind of stress, which in turn can trigger Facebook addiction (Oldmeadow et al., 2013). Life stress has also been found to conduce to WeChat addiction (Li et al., 2018). Interpersonal relationship stress is characterized by a high frequency of occurrence and positively associated with Internet addiction (Yan et al., 2014). Among various types of life stress, interpersonal relationship stress exerts the greatest impact on smartphone addiction (Chiu, 2014). The desire to maintain interpersonal relationships is one of the primary goals of Facebook use (Kwak et al., 2014) and is, in turn, positively related to Facebook addiction (Tang et al., 2016). We hypothesize, then, that WeChat use serves as a way to relieve negative emotions and experiences in interpersonal relationships. Accordingly, our first hypothesis is stated as follows: Interpersonal relationship stress is positively associated with WeChat addiction (H1).

Prior research has demonstrated a positive association between the number of stresses people experience and the amount of time they spend on Internet (Kraut et al., 1998). In the context of SNS, specifically, interpersonal relationship difficulties contribute to higher levels of Facebook use (Elphinston and Noller, 2011), uncertainty reduction theory suggests that the use intensity on Facebook is predicted by undergraduate users’ perceived mutual and definitional uncertainty in a romantic relationship (Stewart et al., 2014), and WeChat use is a measure to relieve undergraduate’s negative emotions and experiences caused by life stressors (Li et al., 2018). Users may also be more likely to use WeChat if they suffer from severer interpersonal relationship stress.

People often use SNS in a habitual way, thereby engaging in frequent and enduring online activities. For instance, the frequency of online communication (Yang et al., 2016) and the time spent online (Wu et al., 2013) are positively related to SNS addictive tendencies. In the context of Facebook, the more one uses the platform, the more likely the user is to develop Facebook addiction (Błachnio et al., 2016). In a similar vein, a WeChat user may gradually seek more and more online intense stimulation, and the initial use intensity might not be sufficient at a later stage.

Based on the above literature review on the expected associations between interpersonal relationship stress and WeChat use intensity, and between WeChat use intensity and WeChat addiction, our second hypothesis is stated as follows: WeChat use intensity mediates the positive association between interpersonal relationship stress and WeChat addiction (H2).

Online social interaction is defined as the degree to which SNS are perceived as communication platforms to interact with others (Cao et al., 2020). The desire for social interaction is an essential human motive for using electronic media in the information age and, moreover, progressively increasing levels of online interaction and information exchange are vital for the meaningful development of interpersonal relationships (Acquisti et al., 2015). SNS are convenient platforms for having social interaction, which does not involve face-to-face communication (Tang et al., 2016). And stress relief is readily elicited in the experience of online interaction between SNS users (Wang and Lee, 2020). Thus, interpersonal relationship stress is positively related to SNS social interaction (Elphinston and Noller, 2011). Similarly, WeChat social interaction offers a high level of privacy and even anonymous, thereby lowering users’ social inhibitions (Chen et al., 2017). It is not surprising to find that WeChat is one of the most popular SNS for engaging in online social interaction (Chen et al., 2017). Accordingly, we expect that interpersonal relationship stress is associated with WeChat social interaction.

SNS enable higher levels of technology-supported interactions and have brought about multiple changes in social interactions (Kaur et al., 2021). SNS social interaction facilitates the formation and maintaining of social relationships among users and urges them to check the online status of their friends repeatedly (Wang and Wang, 2013). And interactivity is one of the incentive stimuli leading to compulsive mobile SNS use (Wang and Lee, 2020). Thus, online social interaction is found to be positively associated with SNS addiction (Yang et al., 2016). Similarly, WeChat social interaction has been empirically demonstrated to be related to excessive use of WeChat (Hou et al., 2017; Cao et al., 2020).

Based on the above literature review on the expected associations between interpersonal relationship stress and WeChat social interaction, and between WeChat social interaction and WeChat addiction, our third hypothesis is stated as follows: WeChat social interaction mediates the positive association between interpersonal relationship stress and WeChat addiction (H3).

In terms of time spent online, the highest ranked motive for Chinese college students is mobile social interaction, followed by mobile video and phone game (QuestMobile, 2019). And WeChat is the most favorite platform to fulfill online social interaction in China (Chen et al., 2017). WeChat users also tend to transfer their offline social interaction with friends to online environment (Hou et al., 2018). In this way, we expect a link between WeChat use intensity and social interaction.

Based on the above literature review on the expected mediating roles of WeChat use intensity (H2) and social interaction (H3), and the expected association between these two mediators, our hypothesis of a three-path mediating effect is stated as follow: WeChat use intensity and social interaction sequentially mediate the positive association between interpersonal relationship stress and WeChat addiction (H4).

In an attempt to investigate whether and how interpersonal relationship stress influences WeChat addiction, the current study proposed a multiple mediating model and examined the mediation hypotheses using bootstrapping methods (Preacher and Hayes, 2008). In particular, we posited that WeChat use intensity and WeChat social interaction sequentially play a mediating role in the association between interpersonal relationship stress and WeChat addiction. Establishing these relationships will lead to a more refined understanding of WeChat.

**FIGURE 1:**
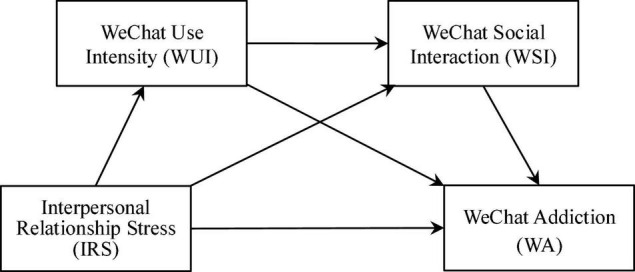
The hypothesized model showing the four hypotheses: H1 (IRS→WA), H2 (IRS→WUI→WA), H3 (IRS→WSI→WA), and H4 (IRS→WUI→WSI→WA).

The hypothesized model of the present study is shown in Figure 1.

## Materials and Methods

### Participants

Data were collected from 463 voluntary undergraduates in southern mainland China by random cluster sampling, who anonymously completed a paper-and-pencil questionnaire in a formal classroom setting in a 20-min time slot, and were informed that their self-report data would be used only for statistical analyses in scholarly articles. The sampling is adequate for the present study following the widely accepted rules on sample size suggested by Nunnally (1994). The respondents were active WeChat users between the age of 17 and 23 (*SD* = 0.98, *M* = 19.12). Two hundred and sixteen respondents were men, and 247 were women; 34.3% were freshmen, 33.9% sophomores, 22.9% juniors, and 8.9% seniors. Ethics approval has been achieved from the responsible Human Subjects Review Committee. All national regulations and laws regarding human participants research were followed.

### Measures

The Adolescent Interpersonal Relationship Stress Scale (AIRSS), a subscale of the Adolescent Self-rating Life Events Checklist developed by Liu et al. (1997), was used to measure the participants’ interpersonal relationship stress. It includes five items (e.g., “Being misunderstood”). In accordance with Tang et al. (2014) and Li et al. (2018), AIRSS was used as a 6-point Likert-type scale to measure the participants’ subjective suffering caused by interpersonal relationship problems during the past year. If a participant answered “no” to an item, the score was 0. If a participant answered “yes,” he/she rated the degree of the stress, yielding a score ranging from 1 (not at all) to 5 (very much). A higher total score on the AIRSS means more severe interpersonal relationship stress experienced in the past year. Cronbach’s alpha of AIRSS in the current study was 0.749.

The 5-point Likert-type Intensity of WeChat Use Scale (IWUS), including four items developed by Li et al. (2019), was used to gauge participants’ WeChat use intensity. The IWUS focused on behavioral indicators such as the duration and the frequency of WeChat use (e.g., “Approximately how many times do you log onto WeChat per day?”). Cronbach’s alpha of IWUS in the present study was 0.716.

The 5-point Likert-type WeChat Social Interaction Scale (WSIS), was developed to measure WeChat social interaction. The scale includes three items: “I have used WeChat to look up someone I met socially,” “I use WeChat to learn more about other people in my classes,” and “I use WeChat to keep in touch with my old friends.” Cronbach’s alpha of WSIS in the current study was 0.621.

The 5-point Likert-type WeChat Addiction Scale (WAS), including five items (e.g., “I feel despaired when I haven’t logged onto WeChat for 1 day”) developed by Li et al. (2018), was used to measure respondents’ WeChat addiction. WAS yields rates that indicate the severity of excessive involvement in WeChat activities, higher scores showing more severe dependence. Cronbach’s alpha of WAS in the present study was 0.815.

### Statistical Analyses

Two preliminary analyses were run, followed by hypotheses examination. First, descriptive statistics and correlation between the research variables were investigated. Second, the normality of data was tested based on the acceptable range on sample distribution [i.e., absolute skewness < 2 and absolute kurtosis < 7 (West et al., 1995)].

In examining the aforementioned hypotheses in Figure 1, the maximum likelihood (ML) approach was utilized to calculate the parameters of the hypothesized model. In accordance with Kwak et al. (2014), the indirect effects of interpersonal relationship stress on WeChat addiction were examined using the bootstrapping method (Preacher and Hayes, 2008). Both CFA and the hypotheses examination were carried out with structural equation modeling (SEM) using Mplus version 7. Following Kline (2010), findings from SEM analysis were assessed by examining multiple fit indices: Chi-square, Tucker-Lewis index (TLI), comparative fit index (CFI), and root-mean-square error of approximation (RMSEA). Conventional guidelines suggest that TLI, CFI ≥ 0.90 indicate adequate model fit, while RMSEA values ≤ 0.08 indicate acceptable model fit and ≤ 0.05 indicate good model fit.

## Results

### Preliminary Analyses

The results in Table 1 shows that Chinese undergraduates tended to report trivial interpersonal relationship stress (1.00 ± 0.83). Interpersonal relationship stress significantly correlated to WeChat use intensity (*r* = 0.141, *p* < 0.01) and WeChat addiction (*r* = 0.106, *p* < 0.05). Findings from these analyses also demonstrated that Chinese undergraduates reported moderate levels of WeChat use intensity (3.12 ± 0.88), WeChat social interaction (3.46 ± 0.79), and WeChat addiction (3.17 ± 0.80). WeChat use intensity significantly correlated to WeChat social interaction (*r* = 0.238, *p* < 0.01) and WeChat addiction (*r* = 0.425, *p* < 0.01). There was also a significant association between WeChat social interaction and WeChat addiction (*r* = 0.495, *p* < 0.01).

**TABLE 1:** Descriptive, pearson correlations, and normality for all variables.

Variables	*M* ± *SD*	Min	Max	Skewness	Kurtosis	1	2	3
1. Interpersonal relationship stress	1.00 ± 0.83	0	4	1.02	0.74	1		
2. WeChat use intensity	3.12 ± 0.88	1	5	–0.19	–0.66	0.140**	1	
3. WeChat social interaction	3.46 ± 0.79	1	5	–0.78	0.79	0.049	0.238**	1
4. WeChat addiction	3.17 ± 0.80	1	5	–0.31	0.13	0.106*	0.425**	0.495**

*p < 0.05; **p < 0.01.

*M, mean; SD, standard deviation.*

The values of research variables’ skewness and kurtosis (see Table 1) showed that they were relatively normally distributed.

### Hypotheses Examination

The independent variables of the hypothesized model comprised three predictor variables: interpersonal relationship stress, WeChat use intensity, and WeChat social interaction. The mediatory variables included WeChat use intensity and WeChat social interaction. And the outcome variable was WeChat addiction. The goodness-of-fit of the hypothesized model is satisfactory: χ^2^(113) = 316.675, *p <* 0.001; CFI = 0.910; TLI = 0.901; RMSEA = 0.062.

**FIGURE 2:**
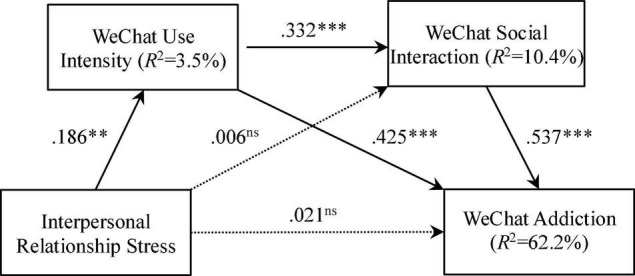
Conceptual model of the standardized direct associations between the research variables. *^ns^*not-significant; ^**^p < 0.01; ^***^p < 0.001.

The total effect of interpersonal relationship stress on WeChat addiction is presented in Table 2. Following the suggestion of Malhotra et al. (2014), both the size and confidence interval of the mediated effects were reported along with their statistical significance, and the indirect effects were specified and contrasted with the mediators (see Table 2). And the standardized direct effects between the four research variables are presented in Figure 2.

**TABLE 2:** Standardized estimates from the structural model.

Effects	Path	Coefficient	Confidence intervals*^pb^*	Test results
Total effects	IRS→WA	0.133*		H1 Accepted
Direct effects	IRS→WA	0.021^ns^		
	IRS→WUI	0.186**		
	IRS→WSI	0.006^ns^		
	WUI→WSI	0.322***		
	WUI→WA	0.425***		
	WSI→WA	0.537***		
Mediated effects	IRS→WUI→WA	0.079**	[0.019, 0.139]	H2 Accepted
	IRS→WSI→WA	0.003*^ns^*	[−0.073, 0.079]	H3 Rejected
	IRS→WUI→WSI→WA	0.032*	[0.006, 0.059]	H4 Accepted

*p < 0.05; **p < 0.01; ***p < 0.001; ^ns^non-significant; ^pb^percentile bootstrap 95%; IRS, interpersonal relationship stress; WA, WeChat addiction; WUI, WeChat use intensity; WSI, WeChat social interaction.

As shown in Table 2 and Figure 2, interpersonal relationship stress exerted a significant total effect on WeChat addiction (β = 0.133, *p <* 0.005) and a direct effect on WeChat use intensity (β = 0.186, *p <* 0.05), thereby enabling H1 to be accepted. However, interpersonal relationship stress had no significant direct effect on WeChat social interaction (β = 0.006, *p* = 0.938), or on WeChat addiction (β = 0.021, *p* = 0.685). WeChat use intensity was significantly and directly associated with WeChat social interaction (β = 0.322, *p <* 0.001) and WeChat addiction (β = 0.425, *p <* 0.001). In addition, WeChat social interaction had a significant direct effect on WeChat addiction (β = 0.537, *p <* 0.001). Finally, interpersonal relationship stress accounted for 3.5% of the variance in WeChat use intensity; interpersonal relationship stress and WeChat use intensity accounted for 10.4% of the variance in WeChat social interaction. Furthermore, interpersonal relationship stress, WeChat use intensity, and WeChat social interaction together accounted for 62.2% of the variance in WeChat addiction.

After mediators were introduced, interpersonal relationship stress showed no significant direct effect on WeChat addiction (see Table 2). According to the findings of Zhao et al. (2010), this indicates that the mediators fully mediated the contribution of interpersonal relationship stress on WeChat addiction (β = 0.111, *p <* 0.01). The significant indirect effects of WeChat use intensity and WeChat social interaction accounted for 83.46% of the total effect, following two paths: (a) WeChat use intensity mediated the association between interpersonal relationship stress and WeChat addiction (β = 0.079, *p <* 0.01). WeChat use intensity’s indirect effect accounted for 59.40% of the total effect of interpersonal relationship stress on WeChat addiction, thereby enabling H2 to be accepted; (b) WeChat use intensity and WeChat social interaction sequentially mediated the association between interpersonal relationship stress and WeChat addiction (β = 0.039, *p <* 0.05). The indirect effect via WeChat use intensity and WeChat social interaction accounted for 24.06% of the total effect, thereby enabling H4 to be accepted. In addition, WeChat social interaction showed no significant mediational effect on the association between interpersonal relationship stress and WeChat addiction (β = 0.003, *p* = 0.938), causing us to reject H3.

Adding gender as a control variable in the hypothesized model, the structural model analysis result is described in Table 3, in which the test results of total effects and mediated effects are the same as those in Table 2.

**TABLE 3:** Standardized estimates from the structural model controlling for gender.

Effects	Path	Coefficient	Confidence intervals*^pb^*	Test results
Total effects	IRS→WA	0.133*		H1 accepted
Direct effects	IRS→WA	0.031^ns^		
	IRS→WUI	0.194**		
	IRS→WSI	0.013^ns^		
	WUI→WSI	0.332***		
	WUI→WA	0.308***		
	WSI→WA	0.525***		
Mediated effects	IRS→WUI→WA	0.079**	[0.031, 0.145]	H2 accepted
	IRS→WSI→WA	0.007*^ns^*	[−0.072, 0.078]	H3 rejected
	IRS→WUI→WSI→WA	0.031*	[0.011, 0.065]	H4 accepted

*p < 0.05; **p < 0.01; ***p < 0.001; ^ns^non-significant; ^pb^percentile bootstrap 95%; IRS, interpersonal relationship stress; WA, WeChat addiction; WUI, WeChat use intensity; WSI, WeChat social interaction.

## Discussion

Due to the high prevalence of WeChat use among Chinese undergraduate students, in particular, WeChat addiction among this population is becoming an increasingly common phenomenon (Gong et al., 2019). There are increasing basic and clinical research on the link between stressful life events and the overuse of WeChat (Andreassen and Pallesen, 2014; Li et al., 2018). The current study sought to untangle how life stress could cause the addictive behavior on WeChat, pinpoint the specific type of stressor, and identify mediating mechanisms in development of the behavioral condition.

### Trigger Role of Interpersonal Relationship Stress

Stress is often perceived in maintaining interpersonal relationship. As predicted, stress from interpersonal relationship brings on WeChat addiction. Life stress has been illustrated to drive the excessive use of WeChat (Li et al., 2018). The present study empirically pinpoints the significance of considering the role of WeChat users’ interpersonal relationship stress in further unraveling the psychological mechanism. This finding is supported by previous studies, which have demonstrated a positive association between interpersonal relationship stress and various forms of cyberaddiction, such as Internet addiction (Seo et al., 2009; Tang et al., 2014), Facebook addiction (Tang et al., 2016), and mobile phone addiction (Chiu, 2014). Interpersonal relationship stress leads to negative emotions, and undergraduates who experience such stress often feel more comfortable in cyberspace and spend excessive time on WeChat to alter the aversive emotional state. The descriptive analyses show that the participants suffered trivial interpersonal relationship stress (1.00 ± 0.83). It is reasonable that its significant total effect on participants’ WeChat addiction was only 0.133.

### Mediating Mechanisms Underlying WeChat Addiction

We identified two mediating mechanisms that underlie interpersonal relationship stress and WeChat addiction. WeChat use intensity mediates the association between interpersonal relationship stress and WeChat addiction. In the first stage, interpersonal relationship stress leads to WeChat use intensity, corroborating the compensatory internet use theory, which concludes that life stresses can motivate individuals to seek psychological comfort via cyberspace (Kardefelt-Winther, 2014). This finding is in line with the conclusion of Tang et al. (2016) who have demonstrated a positive association between interpersonal relationship stress and SNS use. In the second stage, WeChat use intensity is positively associated with WeChat addiction. If WeChat users are more and more involved in WeChat, they are more likely to develop an addiction to WeChat. This is consistent with previous studies that reveal a positive association between use intensity and cyberaddiction (Błachnio et al., 2016; Yang et al., 2016). The availability of fast and cheap Internet access in China makes it easier to use WeChat (Li et al., 2019). The current analyses show that the more undergraduates use WeChat the more inclined they would be to develop WeChat addiction. It is not surprising that WeChat use intensity accounted for 59.40% of the total effect of participants’ interpersonal relationship stress on WeChat addiction.

WeChat use intensity and WeChat social interaction sequentially play a mediating role in the association between interpersonal relationship stress and WeChat addiction. In the first stage, interpersonal relationship stress is positively associated with WeChat use intensity. In the second stage, WeChat use intensity is positively related to WeChat social interaction. Computer-mediated communication is easier, less risky, and more exciting than face-to-face communication (Shaw and Black, 2008), and so it makes sense that the majority of Chinese undergraduates prefer to engage in social interaction on WeChat (QuestMobile, 2019). The third stage of the link between WeChat social interaction and WeChat addiction confirms prior studies, which indicate the positive relationships between online social interaction and Internet addiction (Wang et al., 2015) and SNS addiction (Yang et al., 2016; Hou et al., 2017; Gong et al., 2019; Cao et al., 2020). Our analyses show that participants’ interpersonal relationship stress and the two mediators accounted for 62.2% of the variance in WeChat addiction, and 83.46% of the total effect. Moreover, interpersonal relationship stress affected WeChat addiction only indirectly after the two mediators were introduced.

### Application of Results

The significant trigger role of interpersonal relationship stress behooves college administrative offices to provide solutions to the specific issue in order to reduce undergraduate students’ WeChat addiction. Possible effective measures include cognitive-behavioral group counseling and peer counseling training programs, which have been demonstrated to be beneficial for alleviating interpersonal relationship stress among Internet addicts (Eun, 2002). It may also be helpful for undergraduates with interpersonal relationship stress to communicate more with their families, as researchers have identified a favorable association between family communication time and interpersonal problems (Seo et al., 2009).

Considering its mediatory role in the association between interpersonal relationship stress and WeChat addiction, WeChat use intensity is crucial in the development of WeChat addiction. Users should be discouraged from the frequent and routine WeChat use at school and in the workplace. It is advisable to monitor WeChat use intensity in the design of strategies to prevent WeChat addiction.

Functioning as the second mediator in the three-path mediating effect (see Figure 2), WeChat social interaction is also critical to the development of addiction to WeChat. Expectations relevant to the Internet are positively associated with attitudes toward online social interaction (Lee et al., 2015). Thus, our findings suggest that serious attention should be paid to changing WeChat users’ expectations of WeChat by pointing out the detrimental effects, such as unhealthy lifestyles and poor time management, that accompany excessive use of WeChat when designing interventions for undergraduates with WeChat addiction.

### Conclusion and Future Research Directions

The present study empirically revealed that users’ interpersonal relationship stress significantly leads to WeChat addiction, and that their accumulation of stress in interpersonal relationships gives rise to the intensity of WeChat use, which in turn fuels rising addiction to WeChat both directly and indirectly via social interaction on WeChat. In addition, these findings are robust to controlling for gender.

The empirical research only presents preliminary conclusions on how stressful life events could cause the addictive behavior on WeChat. Certain limitations of the current study have to be acknowledged. This work is a cross-sectional study, and a longitudinal study may be more valid and reliable for measuring interpersonal relationship stress and WeChat addiction, which are dynamic variables that are likely to vary at different points in time. Future research may employ a longitudinal study with multiple measurement points in time to delve into the underlying mechanism of SNS addiction (Wang et al., 2015). Second, all measures in the present study are based on undergraduates’ self-reporting. Future researches considering the associations between interpersonal relationship stress and cyber behavior would benefit from using multiple data collection methods simultaneously in order to produce more objective and complete data. For example, the hair cortisol maybe serve as an objective indicator of interpersonal relationship stress (Schreier et al., 2016), and SNS users’ behavior can be assessed collecting passive data through application-programming interface (Marengo et al., 2020). Furthermore, our criteria for WeChat addiction were adopted mainly based on the obsessive-compulsive foundation. Further studies could establish more clear-cut criteria for diagnosing WeChat addiction.

## Data Availability Statement

The raw data supporting the conclusions of this article will be made available by the authors, without undue reservation.

## Ethics Statement

The studies involving human participants were reviewed and approved by the Human Subjects Review Committee. Written informed consent for participation was not required for this study in accordance with the national legislation and the institutional requirements.

## Author Contributions

BL was involved in study design, implementation, and write-up. KZ was involved in data analysis and write-up. YW collected and analyzed the data. ZH was involved in study design and theorizing. All authors contributed to the article and approved the submitted version.

## Conflict of Interest

The authors declare that the research was conducted in the absence of any commercial or financial relationships that could be construed as a potential conflict of interest.

## Publisher’s Note

All claims expressed in this article are solely those of the authors and do not necessarily represent those of their affiliated organizations, or those of the publisher, the editors and the reviewers. Any product that may be evaluated in this article, or claim that may be made by its manufacturer, is not guaranteed or endorsed by the publisher.
